# Surgery versus sclerotherapy versus combined therapy in head and neck lymphatic malformations in the pediatric population: systematic review and meta-analysis

**DOI:** 10.1007/s00405-024-08661-6

**Published:** 2024-05-07

**Authors:** Jurriën L. A. Embrechts, Steven Hiddinga, Joseph C. Bot, Jan-Jaap Hendrickx, Rik van Eekelen, Johannes C. F. Ket, C. René Leemans, Remco de Bree

**Affiliations:** 1 Department of Otolaryngology-Head and Neck Surgery, Amsterdam UMC, Location VUmc, Amsterdam, The Netherlands; 2 Department of Otolaryngology-Head and Neck Surgery, Christchurch Public Hospital, Christchurch, New Zealand; 3 Department of Radiology and Nuclear Medicine, Amsterdam UMC, Location VUmc, Amsterdam, The Netherlands; 4 Medical Library, Vrije Universiteit, Amsterdam, The Netherlands; 5 Epidemiology and Data Science, Amsterdam UMC, Location VUmc, Amsterdam, The Netherlands; 6 Department of Head and Neck Surgical Oncology, University Medical Center Utrecht, Utrecht, The Netherlands

**Keywords:** Lymphatic malformation, Treatment, Head and neck, Pediatric

## Abstract

**Purpose:**

To systematically review current literature on the treatment of lymphatic malformations (LMs) of the head and neck to guide treatment strategy.

**Methods and materials:**

A systematic review and meta-analysis of literature until 16 November 2021 was performed on treatments of LMs in the head and neck.

**Results:**

Out of 9044 articles, 54 studies were eligible for inclusion with 26 studies providing detailed participant data. A total number of 1573 patients with a mean age of 21.22 months were analysed. Comparative meta-analysis did not reveal significant differences two proportions of volume reduction (≥ 50% and 100%) between sclerotherapy and surgical treatment. Regression demonstrated that positive predictors for volume reduction were surgery 17 (95% CI 0.26–34; *p* = 0.047) and treatment of macrocystic lesions 19 (95% CI 5.5–32; *p* = 0.006). Treatment of mixed lesions also demonstrated a trend towards achieving a greater volume reduction (*p* = 0.052). A higher de Serres stage of the lesion had a negative effect on the amount of volume reduction − 3.7 (95% CI − 7.0 to − 0.35; *p* = 0.030).

**Conclusion:**

This comprehensive meta-analysis demonstrated no significant difference in volume reduction between various treatment modalities at study level. However, individual patient data indicated that surgery and larger cyst types are associated with a significant higher percentage of volume reduction, whereas a higher de Serres stage negatively impacted the amount of volume reduction. These findings can be used for patient counseling and treatment planning based on cyst type and de Serres stage. However volume reduction constitutes just one objective within a more complex treatment spectrum.

**Supplementary Information:**

The online version contains supplementary material available at 10.1007/s00405-024-08661-6.

## Introduction

Lymphatic malformations (LMs) are rare slow-flow vascular malformations with an estimated prevalence of 1:4000 births, which commonly occur in the head and neck (48%) and other lymphatic rich areas [3]. The majority (60%) of LMs is observed at birth and 90% before the age of 2 years. Consequently, patients are predominantly young children with both genders being equally affected [8]. LMs can cause significant morbidity in 70% of cases with intermittent swelling (44%), pain (36%), intralesional bleeding (23%), (recurrent) secondary infection (20%), airway compromise (11%), lymphorrhea (6%), cellulitis, cosmetic disfigurement and lymphocytopenia [2].

Infection or intralesional hemorrhage, which is present in 35% of cases, can lead to acute complications [9]. An overall mortality rate of 3.4–5.7% has been reported with LMs [11]. Peri-operative tracheotomy (8.3%) or prolonged endotracheal intubation can be required to ensure safe treatment [16]. LMs in the head and neck can be subdivided in macrocystic (21%), microcystic (24%) and mixed (49%) subtypes, in which occurrence varies along different subsites of the head and neck region. Midline and oral lesions tend to be more microcystic, whereas parotid and submandibular lesions are more often mixed and cervical lesions are predominantly macrocystic and mixed [17]. The term cystic hygroma and lymphangioma refer to macrocystic LM and microcystic LM respectively and should be abandoned as they insinuate a neoplastic origin [18].

LMs may be the result from an aberrant bud arising from a primordial lymph sac and are associated with genetic disorders including trisomies 13, 18 and 21, Noonan syndrome, Turner syndrome, CLOVES syndrome and Klippel-Trenaunay syndrome [8]. In a large number of patients a mutation in the PIK3CA gene, known to play a role in cell growth, isolated in the lymphatic endothelium is found [1]. However the exact pathogenesis of the condition is still unknown.

The management of LMs in the head and neck region is still challenging and requires a multi-disciplinary team of head and neck surgeons, interventional radiologists and maxillofacial surgeons. The principal goal of LM management is restoration or preservation of functional and aesthetic integrity. Spontaneous involution can occur in approximately (3%) but is reported with varying frequency (0–41%) [21].

Various treatment modalities are used to treat this condition, however no consensus exists regarding optimal treatment. Currently, the main treatment options are surgical resection and sclerotherapy or a combination of these, though newer systemic regimens such as the administration of sildenafil are also being ventured [23]. Initially, surgery was the mainstay of treatment. It’s effectiveness lies in removing the totality or subtotality of the cyst(s) and its lining [22]. This can be hampered by the extent of the lesion and trans-spatial growth, which occasionally involves vital structures. As concerns for damage to nervous and vascular structures during surgery rose, sclerotherapy gained popularity among healthcare professionals due to its uncompromising nature of these structures [7]. The effect of sclerotherapy relies firstly on the collapse of the cyst on itself by aspiration and then partly refilling the cavity with a sclerosing agent to initiate an inflammation response of the endothelial lining to ensure fibrosis of the lining on itself, thus eliminating the cavity [22]. Recently, multiple sclerosing agents were trialed in a search for the optimal safety-efficacy profile [24]. Additionally, the potential for recurrent infections, airway compromise, feeding difficulties, interference with the development of normal speech and concerns for aesthetics, complicate treatment planning. De Serres et al. developed a staging system based on location and extension of the LM that helped to predict outcome of surgery. De Serres noted a positive correlation between higher stages and higher complication rate in operated patients, with a 100% risk of complication in with de Serres stage 5 LM [15]. However, this staging system does not take into account the different lesion configurations such as microcystic, mixed, macrocystic, diffuse and focal lesions for each of which management and outcome can differ vastly [25]. The de Serres classification can be found in Table 1. The de Serres staging system combined with cyst typing has been increasingly used. Improvements in imaging possibilities and capabilities aided in more accurate diagnoses of the type and extent of the LM [7]. With reports using improved imaging and more universal staging systems, a better understanding of treatment response can be obtained. Trends become visible showing that, analogous to surgery, macrocystic LMs respond better to sclerotherapy than mixed or microcystic LMs [24].

**Table 1:** De Serres staging

Stage	Location
1	Unilateral infrahyoid
2	Unilateral suprahyoid
3	Unilateral supra- en infrahyoid
4	Bilateral infrahyoid
5	Bilateral supra- en infrahyoid

The most recent systematic review regarding the treatment of LMs in the head and neck region dates from 2012 [26]. Since 2012 combined therapies of sclerotherapy and surgery have been introduced and new research data with often better description of the pathology has appeared [28]. In this systematic review and meta-analysis we compare the effectiveness of three different treatment modalities, sclerotherapy, surgery and sclerotherapy combined with surgery along the cyst type, in an attempt to aid decision-making in treatment for the different types of LMs in the head and neck area.

## Methods

### Selection of studies

A review protocol based on the Preferred Reporting Items for Systematic Reviews and Meta-Analysis (PRISMA) statement (http://www.prisma-statement.org) was used. No ethics committee approval was required. A systematic search on PubMed, Embase.com and Clarivate Analytics/Web of Science Core Collection from inception up to November 2021 (by JLAE, SCH and JCFK). The following terms were used (including synonyms and closely related words) as index terms or free-text words: ‘lymphatic malformation’ and ‘head and neck’. The full search strategy for all databases is available in the supplementary information appendix A. Duplicate articles were excluded. Two authors (SCH, JLAE) independently screened titles and abstracts. We applied the following exclusion criteria: duplicate studies or use of the same dataset, studies not discussing LM, LM not located in the head and neck area (intra-orbital LMs were excluded), absence of cyst type description, treatment modalities other than sclerotherapy or surgery or their combination, venoLMs, animal studies, studies with fewer than 5 cases, cases without intention to treat, studies with a follow-up of less than 0.5 years, and studies discussing EXIT procedures. In cases where abstracts were missing, a full-text review was carried out. After exclusion based on abstracts, both authors independently screened the remaining articles’ full texts. Risk of bias assessment was carried out using the Newcastle–Ottawa quality assessment scale for cohort studies. The scale was modified excluding the comparability follow-up question. Studies with less than 4 points were excluded in addition to studies lacking data on treatment outcome and when treatment results were not traceable to a specific treatment. Studies in English or French were screened by someone fluent in that language, articles in Spanish or Portugese were translated with DeepL version 3.7.277083. Articles in other languages were excluded.

### Data extraction

Data pertaining type of study, location of the institution, treatment goal, population demographics, LM type (microcystic, macrocystic or mixed) and staging (de Serres stage), mediastinal involvement, treatment modality, different sclerosing agents, number of treatments, tracheostomy, adverse events, recurrence rate, follow-up period, treatment outcomes (degree of volumetric reduction, complications, number of treatments needed, average number of treatment) and adjuvant therapy was extracted from the studies.

When available, individual patient level data were extracted for a separate analysis. The de Serres stage and lesion type were noted by the reviewer or inferred from the description whenever possible. Volume reduction was categorised as follows: 1 = 0% reduction, 2 = 1–49% reduction, 3 = 50–74% reduction, 4 = 75–90% reduction, 5 = 100% reduction. In instances where studies described volume reduction qualitatively, we assigned the minimum percentage decrease in volume according to Appendix B. Grading of adverse events was done according to the Clavien-Dindo classification of adverse events [29]. Recurrences were reported when the lesion increased in size after an initial decrease in size post-intervention. In cases where the de Serres staging was reported but treatment outcomes were assigned along different cyst types, the de Serres staging was allocated to the different cyst type subgroups according to their weighted distribution.

### Data analysis

Zotero (version 6.0.26) was used for reference managing. Microsoft Excel 2013 (version 16.72, Microsoft Corp, Redmont, Washington) was used for creating the dataset. R version 4.3.2 (2023-10-31) was used for descriptive statistics and analysis of the data. ANOVA, *t* test, Kruskal–Wallis and Chi-square tests were used to test for significant differences. Confidence intervals will be reported between parentheses throughout the article. We used the meta package for R to conduct a meta-analysis with a random effects model and tested for between-study heterogeneity with the Higgins and Thompson’s *I*^2^ statistic [30]. Meta-analysis were performed on the different cyst types and events of more or equal than fifty percent or hundred percent volume reduction. Volume reduction is quantified as the percentual decrease from the initial volume. To compare the estimates of the different treatments, a fixed-effects meta-regression model was used. Using individual patient data, we estimated associations between patient characteristics and treatment outcome using a one-stage multiple linear regression model to forecast initial lesion volume reduction using the predictors treatment modality, cyst phenotype, and de Serres staging. The reference standards were sclerotherapy, microcystic lesion and de Serres stage one.

## Results

### Literature search

**Fig. 1:**
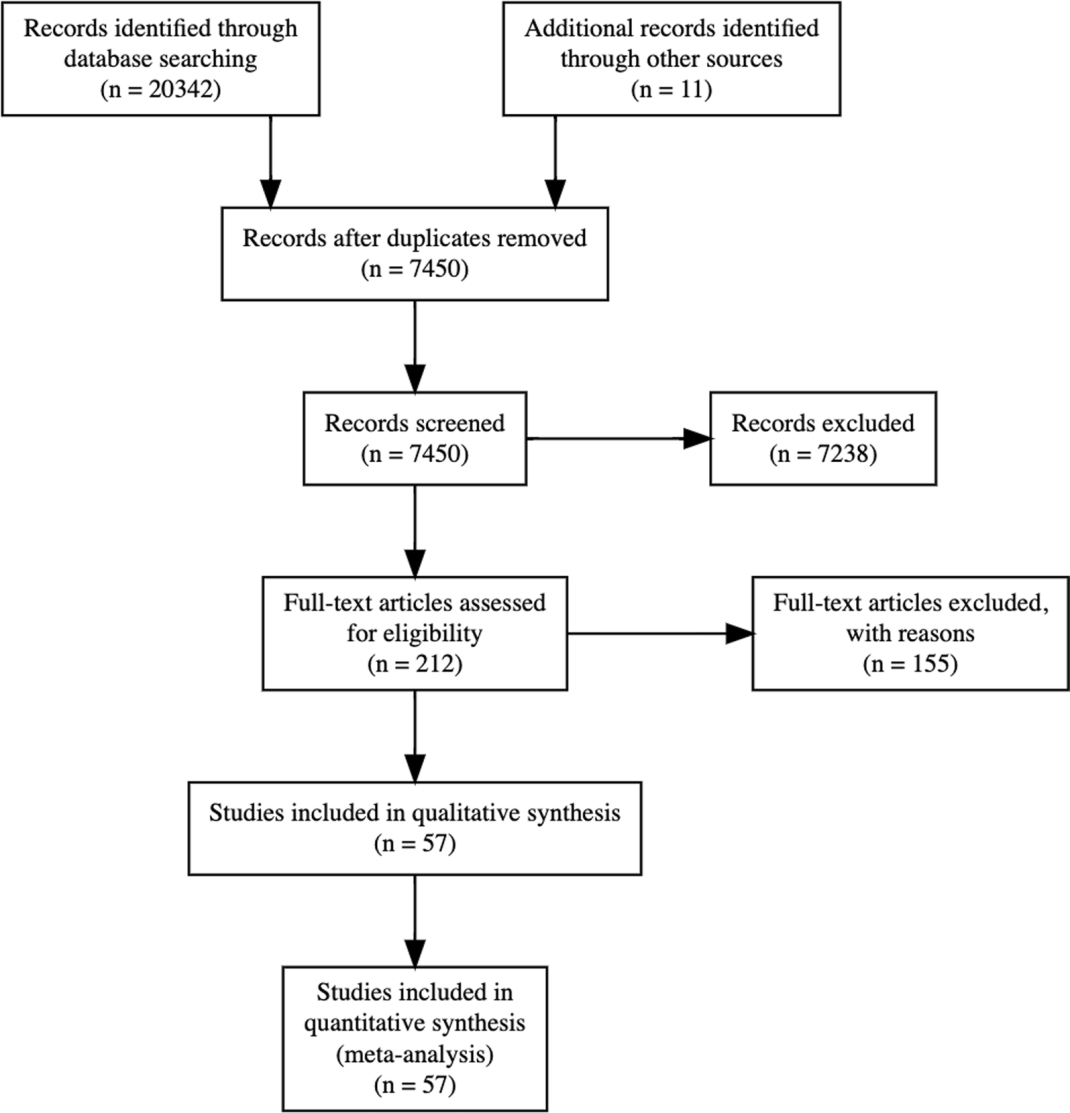
Prisma flowchart

After deduplication of the results, the titles and abstracts of 9,044 articles were screened and 268 were included in the full text review. The full text review resulted in 54 articles eligible for inclusion and 26 articles containing individual-level data. The article selection process is demonstrated according to the PRISMA-statement in the flowchart in Fig. 1. The morphologic description of the malformations was inconsistent between the articles, varying from “invasive”, “cavernous” or “cystic” to absence of lesion description. Consequently, the description of the lesions was used to deduct the lesion type when possible. The resulting number of treated patients with a cyst type description was 1572. A summary of the articles is provided in Table 2.

**Table 2:** Summary of studies

Author	Year	*n*	Modality	Age (months)	Follow-up (months)	Modified Newcastle Ottawa
Tiwari et al.	2020	146	Sclerotherapy	6.0	1.7	6
Wang et al.	2019	128	Surgery	17.0	3.9	6
Smith et al.	2009	115	Sclerotherapy	21.2	2.0	7
Zhi-Min Lei, et al.	2007	89	Surgery	42.0	44.4	5
Wang et al.	2020	72	Sclerotherapy/combined	NA	1.0	6
Ma et al.	2017	68	Surgery	15.0	2.3	5
Yang et al.	2011	65	Sclerotherapy	144.0	1.3	5
Bonilla-Velez et al.	2020	63	Surgery	NA	1.0	5
Zobel, M. J. et al.	2021	63	Sclerotherapy/surgery	16.0	2.3	5
Bajaj et al.	2011	53	Surgery	38.6	2.5	6
Anoop, M et al.	2020	40	Sclerotherapy	82.8	1.0	6
Tu et al.	2017	40	Sclerotherapy	18.5	0.9	4
Gilony et al.	2012	35	Surgery	43.2	2.6	3
Jin et al.	2017	32	Sclerotherapy/combined	24.0	2.4	4
Shiels WE et al.	2009	31	Sclerotherapy	NA	1.0	5
Claesson et al.	2002	29	Sclerotherapy	45.0	2.2	5
Thomas et al.	2016	26	Sclerotherapy	55.8	0.8	5
Kim et al.	2014	26	Sclerotherapy	250.8	1.2	5
Giguere et al.	2002	25	Sclerotherapy	54.0	13.9	5
Weitz-Tuoretmaa et al.	2014	24	Sclerotherapy	21.2	6.0	5
Wittekindt et al.	2006	22	Surgery	83.0	19.9	5
Motz et al.	2014	22	Sclerotherapy	60.0	1.0	5
Upadhyaya et al.	2018	21	Sclerotherapy	NA	0.5	5
Wu et al.	2016	21	Sclerotherapy	75.0	2.8	5
Mirashrafi F. et al.	2021	20	Sclerotherapy	31.9	1.2	5
Luzzatto et al.	2005	20	Sclerotherapy	35.0	0.5	5
Shergill A et al.	2012	19	Sclerotherapy	14.4	1.3	5
Rozman et al.	2011	16	Sclerotherapy	NA	0.9	4
Valletti et al.	2020	15	Sclerotherapy/surgery	32.8	8.0	4
Chen et al.	2017	15	Sclerotherapy	3.0	1.4	5
Chen et al.	2011	14	Sclerotherapy	77.0	1.0	4
Dubois	1997	13	Sclerotherapy	6.0	2.0	6
Karavelioğlu et al.	2010	12	Sclerotherapy	19.5	5.3	5
Greinwald	1999	11	Sclerotherapy	21.7	21.1	5
Sung et al.	1995	10	Sclerotherapy	17.3	1.5	5
Bouatay, R. et al.	2021	10	Sclerotherapy/surgery	28.5	1.1	5
Parashar et al.	2020	10	Sclerotherapy	82.1	4.0	6
Peters et al.	2006	10	Sclerotherapy	45.4	2.0	5
Bonet-Coloma et al.	2011	9	Surgery	73.0	1.1	5
Lee et al.	2016	9	Surgery	35.6	2.0	5
Sichel et al.	2004	8	Sclerotherapy	17.6	2.5	4
Impellizzeri et al.	2010	8	Sclerotherapy	84.0	2.0	5
Shaye et al.	2020	8	Sclerotherapy	19.4	1.7	4
Chen et al.	2020	7	Surgery	46.3	2.6	5
Koo et al.	2016	7	Sclerotherapy	193.7	1.3	5
Benazzou et al.	2013	7	Surgery	72.0	1.2	5
Bhatnagar et al.	2020	7	Sclerotherapy	60.0	2.0	5
Ghaffarpour et al.	2018	7	Surgery/combined	NA	3.5	5
Hassan M. et al.	2020	7	Surgery	30.7	1.0	5
Jamal et al.	2012	6	Sclerotherapy	43.2	2.0	4
Bhatnagar, A. et al.	2020	6	Sclerotherapy	9.5	1.5	5
Gaffuri et al.	2019	6	Surgery	0.5	4.3	5
Wang et al.	2018	5	Sclerotherapy	6.2	1.2	5
Ruiz et al.	2004	NA	Sclerotherapy	37.7	15.9	4

### Design of included articles

The majority 48 out of 54 patients (88.9%) of the included studies with group-level data contained retrospectively collected data while in 3 studies data were prospectively collected. There were 4 controlled studies and 2 studies randomised between a normal and delayed treatment [32]. The studies containing individual patient data were predominantly retrospectively collected case series, with the exception of two studies containing prospectively collected data [33]. Most articles stemmed from a single institutions with multidisciplinary teams as suggested by the fact that the authors were affiliated to different specialisations. Most articles 51 out of 54 patients (94.4%) were published after the year 2000. Tiwari et al. 2020 reported on the largest population (*n* = 146) treated with bleomycin sclerotherapy. Multiple studies reported on more than one treatment modality or compared them. To allow comparison between treatment groups in the group-level data, the population within each study was divided in subgroups along three treatment modalities, sclerotherapy, surgery or combined treatment.

### Demographics

A total number of 1573 patients with a mean age of 21.22 months were treated. The mean follow-up period was 2.00 years and the male to female ratio of 1:1.06. Sclerotherapy was the most frequently used treatment modality 982 out of 1573 patients (62.4%) patients, followed by surgery 501 out of 1573 patients (31.8%) and combined therapy 90 out of 1573 patients (5.7%).

The lesions were predominantly macrocystic 898 out of 1573 patients (57.1%), succeeded by mixed 406 out of 1573 patients (25.8%) and microcystic lesions 269 out of 1573 patients (17.1%). De Serres stage 2 was reported most frequently 488 (42%). The treated LM`s were predominantly macrocystic in the surgery and sclerotherapy group, whereas mixed type lesions were more common in the combined treatment group. The distribution of the lesions along their treatment modality is further described in Table 3.

**Table 3:** Demographics group-level data

Characteristic	Overall, *N* = 1573	Combined, *N* = 90	Sclerotherapy, *N* = 982	Surgery, *N* = 501
Man:woman	1.06	0.76	1.03	1.14
Age (months)	21.22	24.00	21.22	32.80
Follow-up (yrs)	2.00	1.00	1.67	2.60
Lesion type				
Macro	898 (57.1%)	27 (30.0%)	612 (62.3%)	259 (51.7%)
Micro	269 (17.1%)	0 (0.0%)	159 (16.2%)	110 (22.0%)
Mixed	406 (25.8%)	63 (70.0%)	211 (21.5%)	132 (26.3%)
De serres stage				
1	395 (34%)	12 (14%)	213 (36%)	170 (35%)
2	488 (42%)	32 (37%)	247 (42%)	209 (43%)
3	191 (16%)	31 (36%)	98 (17%)	62 (13%)
4	38 (3.3%)	1 (1.2%)	11 (1.9%)	26 (5.4%)
5	46 (4.0%)	10 (12%)	20 (3.4%)	16 (3.3%)
Mediastinum	0.60	1.67	0.33	1.11
Recurrence	0.12	0.14	0.07	0.20
No of treatments (mean)	2.31	1.00	2.83	1.04
No of adjuvant therapy (mean)	0.29	0.17	0.36	0.18
Tracheotomy	0.12	0.21	0.08	0.16
Median; n (%)				

Individual participant data was available in 470 cases. There were no data on combined treatment. The median age was 34 months and median follow-up time was 22 months. In 270 (64%) cases lesions were macrocystic, 89 (21%) were mixed and 62 (15%) were microcystic. The distribution of the cyst types, de Serres stage and follow-up time between the surgery and the sclerotherapy group differed significanty, *p* = 0.003, *p* < 0.001 and *p* < 0.001 respectively. The surgical subgroup treated a higher proportion of microcystic LM with a higher de Serres stage and had a longer follow-up time. An overview is demonstrated in Table 4.

**Table 4:** Demographics individual-level data

Characteristic	Overall, *N* = 470^a^	Sclerotherapy, *N* = 330^a^	Surgery, *N* = 140^a^	*p* value^b^
Sex				0.8
F	225 (48%)	159 (48%)	66 (47%)	
M	245 (52%)	171 (52%)	74 (53%)	
Age (months)	34 (8, 108)	35 (8, 108)	30 (8, 96)	0.4
Unknown	6	1	5	
Lesion type				**0.003**
Macrocystic	270 (64%)	205 (66%)	65 (59%)	
Microcystic	62 (15%)	35 (11%)	27 (25%)	
Mixed	89 (21%)	71 (23%)	18 (16%)	
Unknown	49	19	30	
De serres stage				** < 0.001**
1	177 (41%)	143 (47%)	34 (26%)	
2	153 (35%)	102 (34%)	51 (39%)	
3	64 (15%)	40 (13%)	24 (18%)	
4	17 (3.9%)	4 (1.3%)	13 (9.9%)	
5	23 (5.3%)	14 (4.6%)	9 (6.9%)	
Unknown	36	27	9	
No of treatments	1.00 (1.00, 2.75)	2.00 (1.00, 3.00)	1.00 (1.00, 1.00)	** < 0.001**
Follow-up	22 (11, 30)	15 (8, 28)	30 (18, 30)	** < 0.001**
Volume reduction (%)	95 (52, 100)	90 (52, 100)	100 (69, 100)	** < 0.001**
Tracheostomy	8 (1.7%)	4 (1.2%)	4 (2.9%)	0.2
Recurrence	7 (1.5%)	2 (0.6%)	5 (3.6%)	**0.027**

^a^*n* (%); Median (IQR)

^b^Pearson's Chi-squared test; Wilcoxon rank sum test; Fisher's exact test

The level of significance is: *p* < 0.05

### Treatment outcomes

#### Volume reduction

##### Group-level data

No significant differences were observed in the proportion of lesions achieving ≥ 50% or 100% volume reduction post-treatment across different treatment strategies for all cyst types. The outcomes of the fixed-effects meta-regression model comparing the estimates of surgery and sclerotherapy in achieving ≥ 50% and 100% volume reduction are presented in Tables 5 and 6, respectively. The number of cases receiving combined treatment was insufficient for a meaningful comparison with surgery or sclerotherapy alone.

**Table 5:** Proportion of ≥ 50% volume reduction

Lesion type	Sclerotherapy	Surgery	Overall	*p*
Microcystic	0.68 (0.49–0.82)	0.82 (0.76–0.87)	0.74 (0.63–0.83)	0.083
Mixed	0.8 (0.73–0.86)	0.72 (0.66–0.78)	0.78 (0.72–0.83)	0.120
Macrocystic	0.86 (0.81–0.89)	0.86 (0.8–0.91)	0.86 (0.83–0.88)	0.930

Proportion (95% CI) that achieved ≥ 50% volume reduction compared to the initial lesion

*p* value represents comparison of sclerotherapy and surgery

**Table 6:** Proportion of 100% volume reduction

Lesion type	Sclerotherapy	Surgery	Overall	*p*
Microcystic	0.2 (0.1–0.38)	0.38 (0.13–0.72)	0.27 (0.15–0.43)	0.26
Mixed	0.3 (0.18–0.45)	0.41 (0.32–0.5)	0.39 (0.27–0.53)	0.10
Macrocystic	0.5 (0.35–0.65)	0.66 (0.45–0.82)	0.55 (0.42–0.67)	0.16

Proportion (95% CI) that achieved 100% volume reduction compared to the initial lesion

*p* value represents comparison of sclerotherapy and surgery

For macrocystic lesions, the rates of achieving ≥ 50% volume reduction were similar between surgery and sclerotherapy. However, surgery showed a trend to having a higher proportion of ≥ 50% volume reduction in microcystic lesions (82% vs 68%, *p* = 0.083), while sclerotherapy was more effective in mixed lesions (80% vs 72%, *p* = 0.120). Regarding the proportion of complete (100%) volume reduction, surgery demonstrated a trend to outperforming sclerotherapy across all cyst types. Two studies reported on combined therapy, indicating the highest proportions of 100% volume reduction (73%, range 1–100%) and ≥ 50% volume reduction (96%, range 45–100%) in mixed lesions. Combined therapy was not applied to microcystic lesions.

##### Individual participant data

Table 7 summarises the multiple linear regression model analysing individual participant data. The model identified several key predictors for volume reduction in the treatment of the LM’s compared to the baseline model. Significant positive predictors were: surgery as a treatment modality (*p* = 0.047) and the treatment of macrocystic lesions (*p* = 0.006). Treatment of mixed lesions also demonstrated a trend towards achieving a greater volume reduction (*p* = 0.052). In contrast, a higher de Serres stage negatively impacted volume reduction significantly (p = 0.030).

**Table 7:** Regression model of volume-reduction in individual-level data

Predictors	Estimate^a^	95% CI^b^	*p *value
(Intercept)	69	54, 84	** < 0.001**
Modality			
Sclero	–	–	
Surgery	17	0.26, 34	**0.047**
Lesion type			
Microcystic	–	–	
Mixed	15	− 0.10, 30	0.052
Macrocystic	19	5.5, 32	**0.006**
Sex			
F	–	–	
M	− 0.92	− 7.6, 5.8	0.8
De serres stage	− 3.7	− 7.0, − 0.35	**0.030**
Number of treatments	− 1.8	− 3.9, 0.27	0.088
Study id	− 0.03	− 0.09, 0.02	0.2
Modality × lesion type			
Surgery × mixed	− 13	− 36, 10	0.3
Surgery × macrocystic	2.7	− 16, 21	0.8

^a^Estimates are represented as the percentage of volume reduction of the initial lesion

^b^*CI *confidence interval

The level of significance is: *p* < 0.05

##### Adverse events

Adverse events from the group-level data were categorised according to the Clavien-Dindo grading system presented in Appendix D Table 8. The most frequently observed adverse events were in Clavien-Dindo grade 1 and 4. Grade 1 events such as fever and swelling were notably common across both the combined treatment and sclerotherapy cohorts. Nerve paralysis was observed exclusively in groups undergoing surgery or combined therapies. Specifically, in the surgical cohort, transient facial nerve paralysis was documented in 12 out of 502 patients (2.4%), except for one case with a persisting palsy. The combined treatment group reported 7 out of 90 patients (7.8%) with a transient facial nerve palsy. Other nerve palsies were exclusively reported in the surgical group, marginal mandibular nerve paralysis in 6 out of 502 patients (1.2%) and hypoglossal nerve paralysis in 1 out of 502 patients (0.2%). Infections presented more frequently in the surgical group, affecting 12 out of 502 patients (2.4%), compared to the sclerotherapy group, where they occurred in 3 out of 981 patients (0.3%).

Grade 4 Clavien-Dindo events, including airway obstruction and respiratory failure, were predominantly seen in the sclerotherapy group, with incidences of 10 out of 981 patients (1.0%) and 8 out of 981 patients (0.8%) respectively. In contrast, 27 out of 502 patients (5.4%) received a tracheotomy in the surgical group compared to 5 out of 981 patients (0.5%) in the sclerotherapy group. There were two fatalities in the sclerotherapy group and one in the surgical group, all of which were indirectly linked to the treatments.

In summary, the group-level data showed no significant difference in achieving volume reduction along the various types of LMs between surgery and sclerotherapy. The amount of studies on combined therapy was too low for a meaningful comparison. Analysis of individual participant data with a multiple linear regression model revealed that surgery attained a significantly higher volume reduction compared to sclerotherapy, requiring fewer treatment sessions but associated with a higher rate of symptomatic recurrences. Predictive analysis highlighted surgery and treatment of mixed or macrocystic lesions as positive predictors for volume reduction, while higher de Serres stages negatively impacted the outcome.

Adverse events varied between treatments, with fever and swelling being more common in the sclerotherapy and combined treatment, while infections were more common in surgical cases. Transient nerve palsies were exclusively reported in the surgery and combined treatments groups. Severe complications like airway obstruction were more commonly reported in sclerotherapy, however performing a tracheostomy was more frequent in surgical cases supposedly preventing airway compromise.

## Discussion

This meta-analysis compared different treatment modalities of head and neck LMs. Upon evaluation, there was no significant difference in volume reduction between one of the three therapies (surgery, sclerotherapy or combined) in attaining either hundred percent or more than fifty percent volume reduction. Notably, macrocystic lesions showed the most significant amount of volume-reduction post-treatment, succeeded by mixed and microcystic lesions. These findings are confirmed in previous literature [37]. A lower mean amount of treatment sessions were necessary in the surgical group compared with the sclerotherapy group (*p* < 0.001). Three studies reported on combined treatment, which demonstrated a superior response to therapy in complete as well as partial response in mixed lesions [27]. However this did not significantly differ from other treatment modalities and the number of studies that reported on combined therapy was low. The cases presented in the combined treatment group had an higher mean de Serres stage than the other treatment groups. Previous trends tend to show a less successful outcome with a higher stage [7]. The de Serres stages does not account mediastinal expansion which can influence treatment planning. In a cohort of Ghaffarpour et al. patients requiring emergency surgery presenred with involvement of the mediastinum and/or the abdominal or retroperitoneal cavities [38]. The same author suggested that surgery is the treatment of choice in LM with mediastinal expansion [28]. Respiratory failure and obstruction were observed exclusively after sclerotherapy. This discrepancy might be attributed to surgeons frequently anticipating respiratory complications and consequently employing tracheostomies. Rates of adverse events in surgery such as infection (in 1.5%) [35] conformed as well. Rates of nerve injuries 3.5% and recurrences 7.6% in surgery groups were more prevalent in a surgery group [35] but in other studies non-existent [40]. Fever was reported as an Claviend-Dindo class 1 adverse event, however in sclerotherapy such an event might be expected due to the injection of an inflammatory agent in sclerotherapy.

The meta-analysis conducted in this study carries limitations that merit discussion. First, the principal goal of LM management is restoration or preservation of functional and aesthetic integrity and elimination of objective and subjective symptoms related to the abnormality. In the current literature on head and neck LM, treatment objectives are infrequently documented and solely evaluating volume reduction might not adequately describe treatment success. The majority of the studies lacked a systematic presentation of patient-reported outcomes. Earlier a patient satisfaction of approximately 50–60% was reported after sclerotherapy [41, 42]. Sclerotherapy generally leads to moderate patient-reported improvement in health and quality of life in about half of treated patients, irrespective of the type, size and location of the lesion [43]. Second, the quality of evidence varied among the different studies. No studies featured randomisation between the different treatment modalities and the predominance of the data consisted of restrospectively collected case series. Only 9.1% of included studies were prospective studies, the remaining were retrospective case series. Third, the data was collected from various medical resources and outcomes were measured heterogeneously. Surgery and combined therapy showed the most dramatic response in terms of volume reduction. However, treatment goals between surgery and sclerotherapy might differ. Surgery often aims towards complete excision of the lesion, whereas sclerotherapy is more directed towards volume reduction of the cysts enough to restore anatomical function and relieve subjective symptoms. Contrastingly, in most studies reduction of volume after sclerotherapy was assessed using imaging while surgical outcomes were more often evaluated clinically. Hence, the treatment effect of the surgical groups on quality of life could be overestimated. In the absence of reporting on cyst type, de Serres staging or degree of volume reduction, the outcomes were deduced when possible from the case descriptions in a conservative manner. i.e. when distinction between two de Serres stages was unclear, the lower de Serres stage was reported in our series. Consequently, this could lead to a underestimation of treatment results. In several articles recurrences were reported after sclerotherapy. A clear definition of recurrence was most often not reported and can arguably also be defined as residual lesion. We defined recurrence as an increase in size of the lesions after an initial decrease post-intervention, which followed how this was defined in other articles. In numerous studies, instances of recurrence following sclerotherapy were documented [44]. However, these articles frequently lacked a clear, standardized definition of ‘recurrence’, with some equating it to a ‘residual lesion’. This ambiguity necessitates a clear operational definition, since after sclerotherpy there is always a residual lesion on MR imaging after therapy. Whether there are clinical symptoms or not. For our research purposes, we have characterized ‘recurrence’ as an increase in lesion size following an initial reduction post-intervention, aligning with definitions presented in certain previous studies [46]. Fourth, outcomes could not always be distinguished between the different treatment subgroups and were calculated as weighted means. Consequently, the affected outcomes regress towards the average outcome value of the subgroups combined, potentially obscuring distinct insights. Fifth, the choice of treatment could be influenced by anatomical location and type of the lesion as well as local experience among specialist resulting in a selection bias. It is more likely that lesions most suitable for one of the treatment options (as judged by the treating phsyician) will be treated accordingly. For surgery extension of the lesion is important in decision making, whereas for sclerotherapy it is the cyst type, e.g. macrocystic LM. The aim of the chosen treatment may also differ, complete resection or tumor reduction only. Sixth, the de Serres classification of LM’s was used for subgroup analysis. The de Serres classification does not take into account mediastinal expansion or separate oral cavity analysis which could negatively impact treatment outcome as oral cavity lesions are often micocystic and more prone to obstruct the oral airway. In the individual group most microcystic lesions treated by surgery were located in the tongue.

## Conclusion

This systematic review and meta-analysis included 26 articles covering 1573 patients. Meta-analysis on study level data demonstrated no significant difference in volume reduction between the various treatment modalities. However, when examining individual patient data with a regression using a linear model significant positive predictors for volume reduction were surgery and the treatment of macrocystic lesions compared to sclerotherapy and treating microcystic lesions. Whereas, a higher de Serres stage was negatively associated with the amount of volume reduction. These findings can be used for patient counseling and treatment planning based on cyst type and de Serres stage. However, at an individual level multidisciplinary discussion is necessary taking into account age, clinical presentation, indication and objective.

## Limitations

In the current meta-analysis there has only been comparison between the different treatment modalities on volume reduction. There is still a great heterogeneity reporting the amount of volume reduction. Treatment outcomes like aesthetic or functional integrity, quality of life or PROMS are better aligned with treatment objectives but were seldomly reported.

## Recommendations

Although the paucity of LM’s make a randomised controlled trial challenging, we advocate multi-institutional randomised controlled trial to measuring the effectiveness on the different treatment methods, quality of life assessments and patient reported outcomes. A guideline of documentation of clinical reports should be implemented to ensure homogeneity in documentation and treatment methods.

## Supplementary Information

Below is the link to the electronic supplementary material.

Supplementary file1 (DOCX 1224 KB)

## Data Availability

Our data is available on request to the first author.
