# Supplementary material for: Surgery versus sclerotherapy versus combined therapy in head and neck lymphatic malformations in the pediatric population: systematic review and meta-analysis
Source: Eur Arch Otorhinolaryngol. 2024 May 7;281(9):4529–39. doi: 10.1007/s00405-024-08661-6 (PMC11393230; doi:10.1007/s00405-024-08661-6)
Supplement: Supplementary file 1 — Supplementary file1 (DOCX 1224 KB) [file 405_2024_8661_MOESM1_ESM.docx]

# Supplementary information

Appendix A

## Search strategy for PubMed (16 November 2021)

| Search | Query | Results |
| --- | --- | --- |
| #7 | #6 NOT (animals[mh] NOT humans[mh]) | 6,610 |
| #6 | #3 NOT (#4 OR #5) | 6,747 |
| #5 | (“Brain”[Mesh] OR “Brain Diseases”[Mesh]) | 2,253,856 |
| #4 | (“Hemangioma”[Mesh] OR “Port-Wine Stain”[Mesh] OR hemangiom*[tiab] OR angiom*[tiab]) | 53,647 |
| #3 | #1 AND #2 | 11,379 |
| #2 | (“Esophagus”[Mesh] OR “Ear”[Mesh] OR “Scalp”[Mesh] OR “Head”[Mesh:NoExp] OR “Skull”[Mesh:NoExp] OR “Pharynx”[Mesh] OR “Larynx”[Mesh] OR “Head and Neck Neoplasms”[Mesh] OR “Nose”[Mesh] OR “Laryngeal Mucosa”[Mesh] OR “Nasal Mucosa”[Mesh] OR “Face”[Mesh] OR neck[tiab] OR nasopharyn*[tiab] OR paranasal*[tiab] OR oral*[tiab] OR nose[tiab] OR oesophag*[tiab] OR esophag*[tiab] OR facial[tiab] OR face[tiab] OR eyelid*[tiab] OR mouth[tiab] OR gingiva*[tiab] OR lip[tiab] OR lips[tiab] OR palatal[tiab] OR palate[tiab] OR palatum[tiab] OR salivary[tiab] OR sublingual[tiab] OR submandibular[tiab] OR paroti*[tiab] OR tongue[tiab] OR otorhinolaryn*[tiab] OR ear[tiab] OR ears[tiab] OR laryngeal*[tiab] OR larynx[tiab] OR pharyn*[tiab] OR parathyr*[tiab] OR thyroid*[tiab] OR trachea*[tiab] OR hypopharyn*[tiab] OR oropharyn*[tiab] OR tonsil*[tiab] OR mandib*[tiab] OR subglotti*[tiab] OR trachea*[tiab] OR upper aerodigestive tract*[tiab] OR uadt[tiab] OR oral cavity[tiab] OR nasopharyn*[tiab] OR eyelid*[tiab] OR glotti*[tiab] OR vocal cords[tiab] OR epiglotti*[tiab] OR arytenoid*[tiab] OR cricoid*[tiab] OR thyroid*[tiab] OR nasal*[tiab] OR olfactor*[tiab] OR sinus*[tiab] OR sinonasal*[tiab] OR turbinat*[tiab] OR vomer*[tiab] OR adenoid*[tiab] OR velopharyn*[tiab] OR cheek*[tiab] OR chin[tiab] OR eye[tiab] OR eyes[tiab] OR eyebrow*[tiab] OR forehead[tiab] OR scalp[tiab] OR nasolabia*[tiab] OR paroti*[tiab] OR skull*[tiab] OR cranium*[tiab] OR calvari*[tiab] OR tympan*[tiab] OR auric*[tiab] OR cochlea*[tiab] OR vestibul*[tiab] OR ossicle*[tiab] OR eustachia*[tiab] OR stapedi*[tiab] OR lingual*[tiab]) | 3,203,261 |
| #1 | (“Lymphatic Vessel Tumors”[Mesh] OR ((“Microvessels”[Mesh] OR “Blood Vessels”[Mesh:NoExp] OR “Veins”[Mesh:NoExp] OR “Cerebral Veins”[Mesh] OR “Cranial Sinuses”[Mesh] OR “Vascular Diseases”[Mesh] OR lymphat*[tiab] OR lymphogen*[tiab] OR lymphoven*[tiab]) AND (malformation*[tiab] OR fistula*[tiab])) OR lymphangioma*[tiab] OR lymph angioma*[tiab] OR hygroma*[tiab] OR parkes weber*[tiab] OR klippel-trenaunay*[tiab]) | 56,530 |

## Search strategy for Embase.com (16 November 2021)

| Search | Query | Results |
| --- | --- | --- |
| #6 | #5 NOT ([animals]/lim NOT [humans]/lim) | 11950 |
| #5 | #3 NOT #4 | 12230 |
| #4 | ‘brain’/exp OR ‘brain disease’/exp OR ‘hemangioma’/exp OR ‘nevus flammeus’/exp OR hemangiom*:ti,ab,kw OR angiom*:ti,ab,kw | 3,502,477 |
| #3 | #1 AND #2 | 23,607 |
| #2 | ‘esophagus’/exp OR ‘ear’/exp OR ‘scalp’/exp OR ‘head’/exp OR ‘stomatognathic system’/exp OR ‘larynx’/exp OR ‘head and neck tumor’/exp OR neck:ti,ab,kw OR paranasal*:ti,ab,kw OR oral*:ti,ab,kw OR nose:ti,ab,kw OR oesophag*:ti,ab,kw OR esophag*:ti,ab,kw OR facial:ti,ab,kw OR face:ti,ab,kw OR mouth:ti,ab,kw OR gingiva*:ti,ab,kw OR lip:ti,ab,kw OR lips:ti,ab,kw OR palatal:ti,ab,kw OR palate:ti,ab,kw OR palatum:ti,ab,kw OR salivary:ti,ab,kw OR sublingual*:ti,ab,kw OR submandibular*:ti,ab,kw OR tongue:ti,ab,kw OR otorhinolaryn*:ti,ab,kw OR ear:ti,ab,kw OR ears:ti,ab,kw OR laryngeal*:ti,ab,kw OR larynx:ti,ab,kw OR pharyn*:ti,ab,kw OR parathyr*:ti,ab,kw OR hypopharyn*:ti,ab,kw OR oropharyn*:ti,ab,kw OR tonsil*:ti,ab,kw OR mandib*:ti,ab,kw OR subglotti*:ti,ab,kw OR trachea*:ti,ab,kw OR ((‘upper aerodigestive’ NEAR/3 tract*):ti,ab,kw) OR uadt:ti,ab,kw OR ‘oral cavity’:ti,ab,kw OR nasopharyn*:ti,ab,kw OR eyelid*:ti,ab,kw OR glotti*:ti,ab,kw OR ((vocal NEAR/3 cord*):ti,ab,kw) OR epiglotti*:ti,ab,kw OR arytenoid*:ti,ab,kw OR cricoid*:ti,ab,kw OR thyroid*:ti,ab,kw OR nasal*:ti,ab,kw OR olfactor*:ti,ab,kw OR sinus*:ti,ab,kw OR sinonasal*:ti,ab,kw OR turbinat*:ti,ab,kw OR vomer*:ti,ab,kw OR adenoid*:ti,ab,kw OR velopharyn*:ti,ab,kw OR cheek*:ti,ab,kw OR chin:ti,ab,kw OR eye:ti,ab,kw OR eyes:ti,ab,kw OR eyebrow*:ti,ab,kw OR forehead:ti,ab,kw OR scalp:ti,ab,kw OR nasolabia*:ti,ab,kw OR paroti*:ti,ab,kw OR skull*:ti,ab,kw OR cranium*:ti,ab,kw OR calvari*:ti,ab,kw OR tympan*:ti,ab,kw OR auric*:ti,ab,kw OR cochlea*:ti,ab,kw OR vestibul*:ti,ab,kw OR ossicle*:ti,ab,kw OR eustachia*:ti,ab,kw OR stapedi*:ti,ab,kw OR lingual*:ti,ab,kw | 4,385,067 |
| #1 | ‘lymphangioma’/exp OR ((‘microvasculature’/exp OR ‘blood vessel’/de OR ‘vein’/de OR ‘ophthalmic vein’/exp OR ‘brain vein’/exp OR ‘central vein’/exp OR ‘retina vein’/exp OR ‘cranial sinus’/exp OR ‘vascular disease’/exp OR lymphat*:ti,ab,kw OR lymphogen*:ti,ab,kw OR lymphoven*:ti,ab,kw) AND (malformation*:ti,ab,kw OR fistula*:ti,ab,kw)) OR lymphangioma*:ti,ab,kw OR ‘lymph angioma*’:ti,ab,kw OR hygroma*:ti,ab,kw OR ’parkes weber*’:ti,ab,kw OR ’klippel-trenaunay*’:ti,ab,kw | 99,713 |

## Search strategy for Clarivate Analytics/Web of Science (16 November 2021)

| Search | Query | Results |
| --- | --- | --- |
| #4 | (#1 AND #2) NOT #3 | 1,782 |
| #3 | TS=(“hemangiom*” OR ”angiom*” OR “brain”) | 1,434,179 |
| #2 | TS=((“neck” OR “paranasal*” OR ”oral*” OR “nose” OR “oesophag*” OR ”esophag*” OR “facial” OR “face” OR “mouth” OR “gingiva*” OR ”lip” OR ”lips” OR ”palatal” OR ”palate” OR ”palatum” OR ”salivary” OR ”sublingual*” OR “submandibular*” OR ”tongue” OR ”otorhinolaryn*” OR “ear” OR “ears” OR “laryngeal*” OR ”larynx” OR ”pharyn*” OR “parathyr*” OR ”hypopharyn*” OR “oropharyn*” OR ”tonsil*” OR “mandib*” OR ”subglotti*” OR “trachea*” OR (“upper aerodigestive” NEAR/3 ”tract*”) OR “uadt” OR “oral cavity” OR “nasopharyn*” OR ”eyelid*” OR “glotti*” OR (”vocal” NEAR/3 ”cord*”) OR “epiglotti*” OR ”arytenoid*” OR “cricoid*” OR ”thyroid*” OR “nasal*” OR ”olfactor*” OR “sinus*” OR ”sinonasal*” OR “turbinat*” OR ”vomer*” OR “adenoid*” OR ”velopharyn*” OR “cheek*” OR ”chin” OR ”eye” OR ”eyes” OR ”eyebrow*” OR “forehead” OR “scalp” OR “nasolabia*” OR ”paroti*” OR “skull*” OR ”cranium*” OR “calvari*” OR ”tympan*” OR “auric*” OR ”cochlea*” OR “vestibul*” OR ”ossicle*” OR “eustachia*” OR ”stapedi*” OR “lingual*” OR ((”temporal*” OR “maxillar*” OR ”subclavia*” OR “vertebra*” OR ”brachicephal*” OR “jugular*” OR ”caroti*” OR “innominat*” OR ”occipital*” OR “thyrocervical*” OR ”suprascapular*” OR “cervical*” OR ”angual*” OR “angular*” OR ”retromandibular*” OR “submental*”) NEAR/3 (”artery” OR ”arteries” OR ”arteria*” OR “vena” OR “vein*” OR “venous” OR “truncus” OR “blood vessel” OR “blood vessels”)))) | 3,488,230 |
| #1 | TS=(((“lymphat*” OR ”lymphogen*” OR “lymphoven*”) AND (”malformation*” OR “fistula*”)) OR ”lymphangioma*” OR “lymph angioma*” OR ”hygroma*” OR “parkes weber*” OR ”klippel-trenaunay*”) | 9,480 |

Appendix B: Stratification of qualitative outcomes along their categories

| % Range | 0% | 1-49% | 50-74% | 75-99% | 100% |
| --- | --- | --- | --- | --- | --- |
| Description | none | Slight | Moderate | Nearly complete | Complete cure |
|  | no response | Poor | Intermediate | Excellent | No residual |
|  | unfavorable | Possible | Significant improvement | Subcomplete | Cure |
|  |  | Very little change | Well reduced | Near normal size | Complete |
|  |  | Did not appear significantly reduced | Satisfactory | Marked | Total shrinkage |
|  |  | Fair | Good | Much smaller |  |
|  |  | Somewhat | Well | Mostly |  |
|  |  | Fail |  | Success |  |
|  |  | Partial |  | Substantial |  |
|  |  | Improved |  | Significant |  |
|  |  | Small |  |  |  |
|  |  | Incomplete |  |  |  |
|  |  | Residual disease |  |  |  |

Appendix C:

| 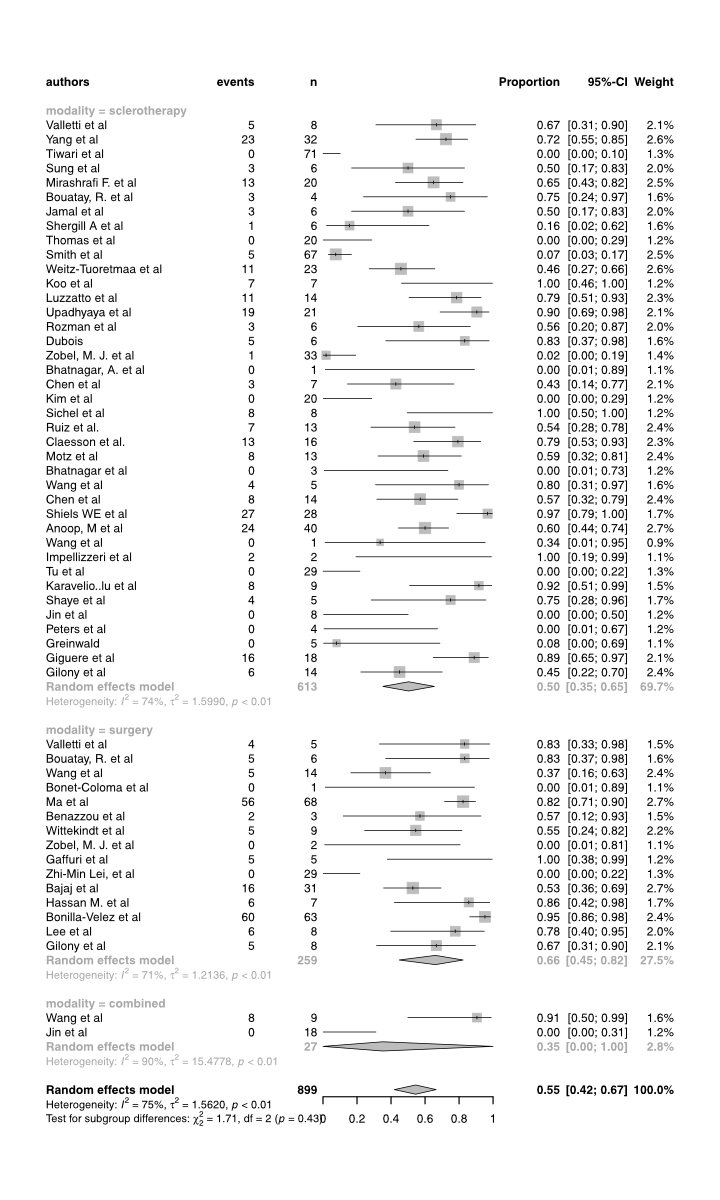  **Table 2** Proportion of 100% volume reduction in macrocystic lymphatic malformations |
| --- |
| 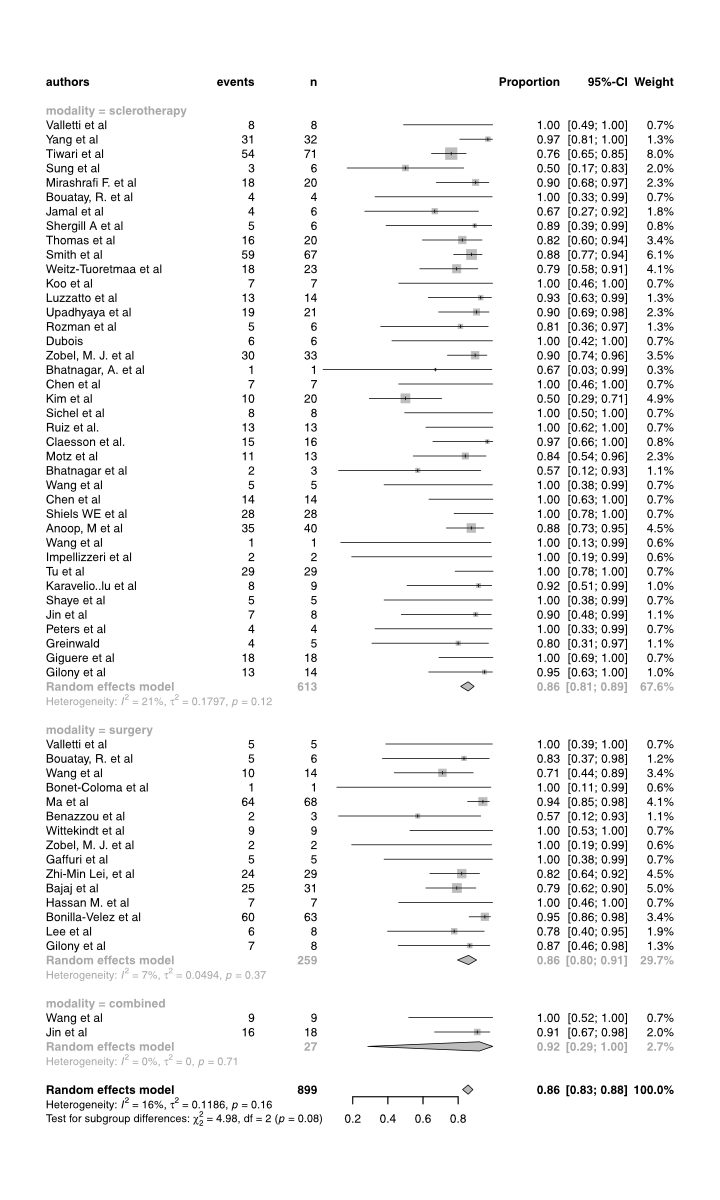  **Table 3** Proportion of >=50% volume reduction in macrocystic lymphatic malformations |

| 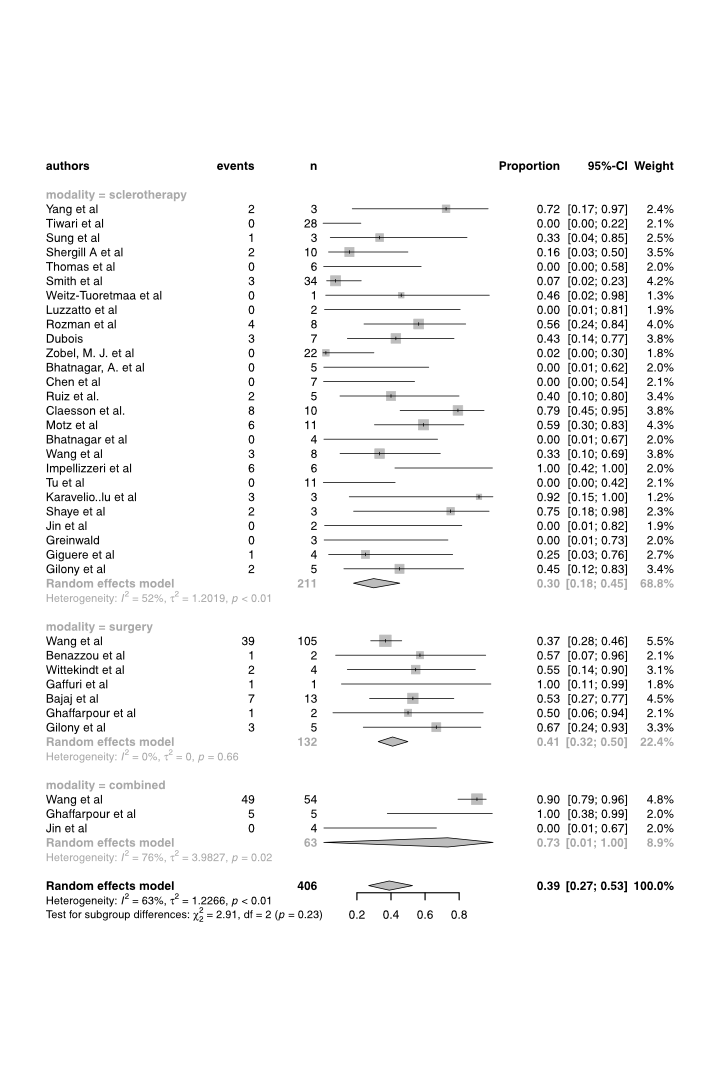  **Table 4** Proportion of 100% volume reduction in mixed lymphatic malformations |
| --- |
| 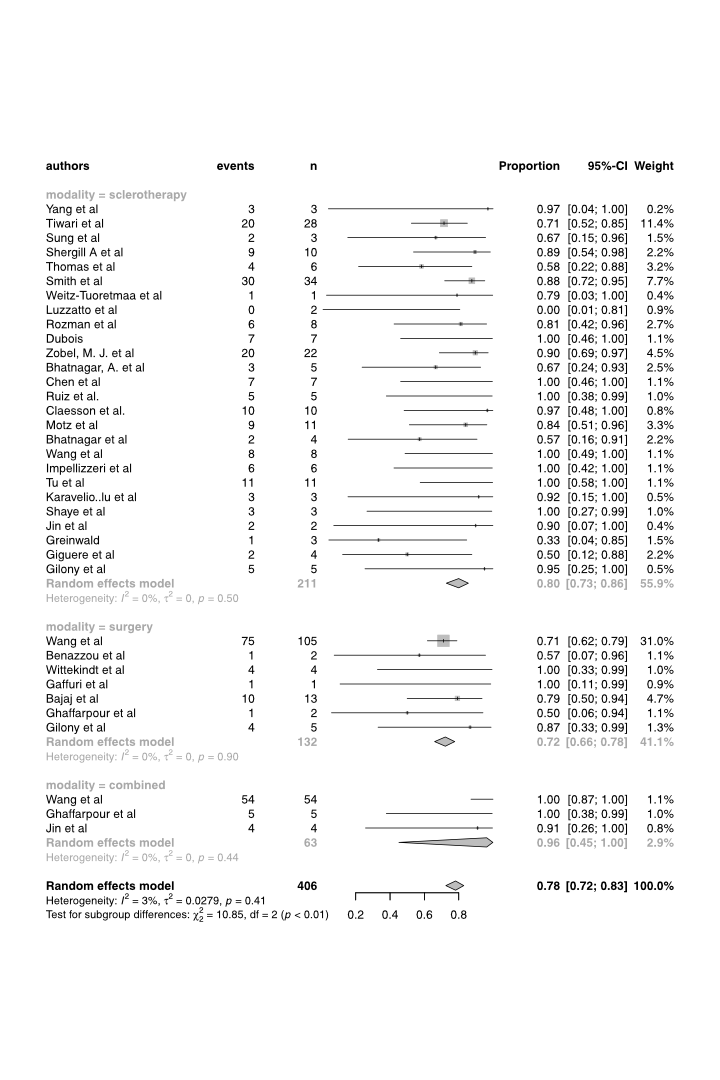  **Table 5** Proportion of >=50% volume reduction mixed lymphatic malformations |

| 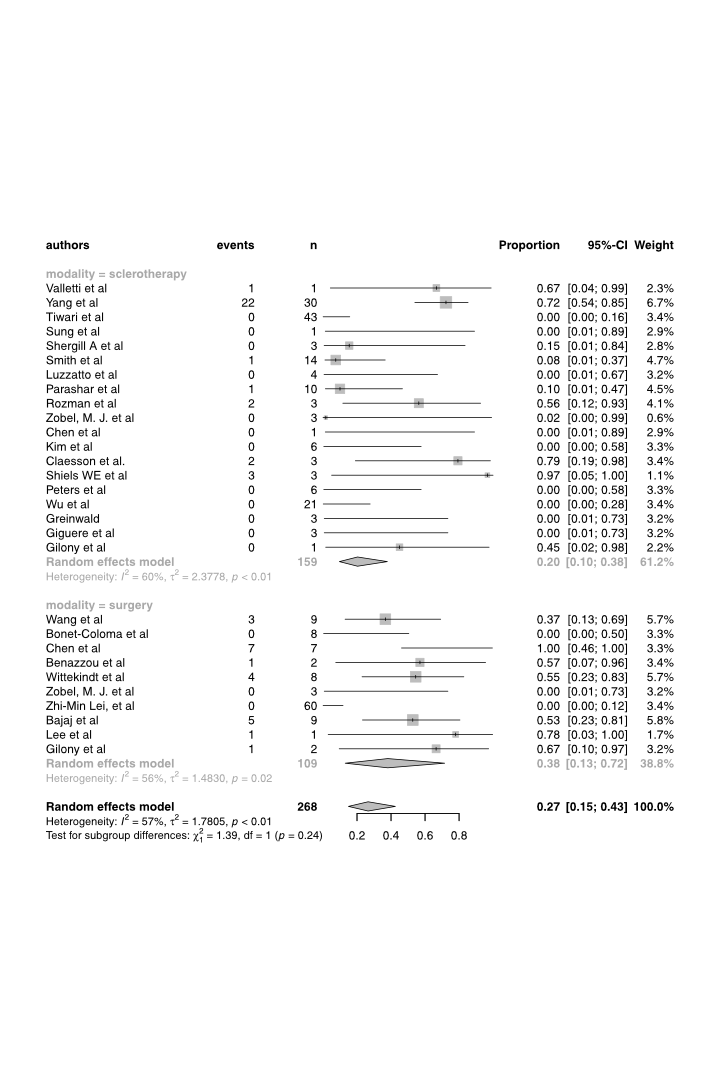  **Table 6** Proportion of 100% volume reduction in microcystic lymphatic malformations |
| --- |

| 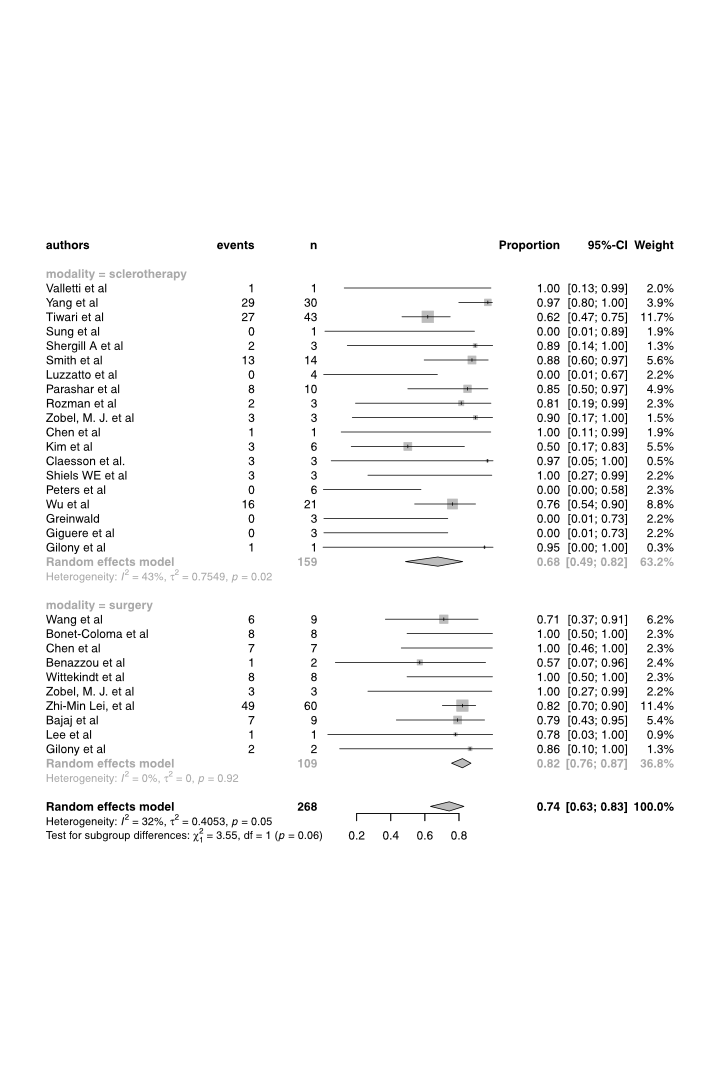  **Table 7** Proportion of >=50% volume reduction microcystic lymphatic malformations |
| --- |

Appendix D

| Event | Combined N=90 | Sclerotherapy N=981 | Surgery N=502 |
| --- | --- | --- | --- |
| **Clavien-Dindo Grade 1** |  |  |  |
| Fever | 18 (20%) | 119 (12.13%) | 0 (0%) |
| Swelling | 23 (25.56%) | 107 (10.91%) | 0 (0%) |
| Erythema | 0 (0%) | 55 (5.61%) | 0 (0%) |
| Facial nerve paralysis | 7 (7.78%) | 0 (0%) | 12 (2.39%) |
| Hypoglossal nerve palsy | 0 (0%) | 0 (0%) | 1 (0.20%) |
| Marginal mandibular nerve paralysis | 0 (0%) | 0 (0%) | 6 (1.20%) |
| Horner’s syndrome | 0 (0%) | 0 (0%) | 1 (0.20%) |
| Cellulitis | 0 (0%) | 5 (0.51%) | 0 (0%) |
| Inflammation | 0 (0%) | 20 (2.04%) | 0 (0%) |
| Hematoma | 0 (0%) | 4 (0.41%) | 14 (2.79%) |
| Anemia | 0 (0%) | 0 (0%) | 0 (0%) |
| Restricted neck movement | 0 (0%) | 0 (0%) | 0 (0%) |
| **Clavien-Dindo Grade 2** |  |  |  |
| Pigmentation | 0 (0%) | 14 (1.43%) | 0 (0%) |
| Atrophy | 0 (0%) | 1 (0.10%) | 0 (0%) |
| Upper airway tract infection | 0 (0%) | 25 (2.55%) | 0 (0%) |
| Fibrotic nodule | 0 (0%) | 1 (0.10%) | 0 (0%) |
| **Clavien-Dindo Grade 3** |  |  |  |
| Pain | 0 (0%) | 45 (4.59%) | 0 (0%) |
| Infection | 0 (0%) | 3 (0.31%) | 12 (2.39%) |
| Ulceration | 0 (0%) | 4 (0.41%) | 0 (0%) |
| **Clavien-Dindo Grade 4** |  |  |  |
| Skinflap necrosis | 0 (0%) | 0 (0%) | 1 (0.20%) |
| Abscess | 0 (0%) | 2 (0.20%) | 0 (0%) |
| Intralesional bleeding | 0 (0%) | 3 (0.31%) | 0 (0%) |
| **Clavien-Dindo Grade 5** |  |  |  |
| Airway obstruction | 0 (0%) | 10 (1.02%) | 1 (0.20%) |
| Respiratory failure | 3 (3.33%) | 8 (0.82%) | 0 (0%) |
| CNS | 0 (0%) | 0 (0%) | 0 (0%) |
| **Other Events** |  |  |  |
| Death | 0 (0%) | 2 (0.20%) | 1 (0.20%) |
| Recurrence | 3 (3.33%) | 16 (1.63%) | 43 (8.57%) |
| Tracheotomy | 3 (3.33%) | 5 (0.51%) | 27 (5.38%) |
| Adjuvant therapy^1^ | 16 (17.78%) | 64 (16.72%) | 39 (27.69%) |
| Exacerbation | 0 (0%) | 0 (0%) | 0 (0%) |
| ^1^ Adjuvant therapy is sclerotherapy after surgery and vice versa |  |  |  |

**Table 8** Adverse events according to the Clavien-Dindo grading system
